# Cancer Care During the COVID-19 Pandemic: The African Narrative and Prospects

**DOI:** 10.7759/cureus.43803

**Published:** 2023-08-20

**Authors:** Charles Ojo, Chijioke Orji, Ayodeji Adedeji, Chibuike Nwachukwu, Ona Fagbemi

**Affiliations:** 1 Emergency Department, United Lincolnshire Hospitals NHS Trust, Boston, GBR; 2 Trauma and Orthopaedics, Betsi Cadwaladr University Health Board, Wrexham, GBR; 3 Emergency Department, Darlington Memorial Hospital, Durham, GBR; 4 Breast Surgery, St George's University Hospitals NHS Foundation Trust, London, GBR; 5 General Surgery, University Hospital North Midlands, Stoke-on-Trent, GBR

**Keywords:** oncology, health care, africa, pandemic, covid-19, cancer care

## Abstract

The COVID-19 pandemic has had a significant impact on healthcare services globally. Whilst it has been particularly disruptive for cancer care in low-resource settings, a few African countries have been able to adapt strategies to enable continued delivery of medical care to persons with cancer. This study seeks to highlight how much effect the coronavirus pandemic has had on oncological care in Africa and to indicate the way forward. For this narrative review, PubMed and Google Scholar were used to search for literature addressing the effect of the coronavirus pandemic on the care of patients with cancer in Africa with ensuing coping strategies. Selection criteria were manuscripts published since the onset of the pandemic in 2019 and written in the English language with Africa being the focus. In total, 52 research papers involving up to 21 African nations were found and reviewed. Across the board, the COVID-19 pandemic resulted in the deferral of oncological screening programs and a halt in immunization activities routinely scheduled for preventable cancers. It caused a colossal shortage in the availability of appropriately trained medical personnel, reduced frequency and duration of outpatient consultations, and a delay in cancer investigations and diagnosis. It also stirred up the substandard modification of chemotherapy regimens and radiotherapy due to the scarcity of anticancer medications and radioisotopes and engendered the cancellation of cancer surgical procedures. Palliative care for patients with locally advanced and metastatic disease was in many cases interrupted and cancer research activities were abruptly deferred. Ultimately, these led to poor patient outcomes and increased cancer-related fatalities. However, a few African countries - Rwanda, Ghana, and Tunisia - have continued to adapt telemedicine, small unmanned aircraft systems (sUAS), and home therapy to facilitate cancer care. To date, there is a paucity of data concerning the successes and cost-effectiveness of these relatively new methods recently adapted to cater to the medical needs of cancer patients in Africa. The pandemic has presented the African community an opportunity to advance her healthcare systems, especially as it pertains to the delivery of medical care to persons with cancer. The need of the hour is to study further the alternative cancer care delivery systems initiated during the pandemic in order to determine their sustainability in Africa at large.

## Introduction and background

Globally, cancer is a leading cause of mortality and, it is estimated that per annum, cancer-related deaths will increase in Sub-Saharan Africa by up to a million by the year 2030 from over 520,000 in the year 2020 [1]. It is a disease that causes body cells to multiply abnormally and uncontrollably with ensuing local invasion and distant metastasis [2]. There are more than 26 distinct types of cancer and they vary substantially in their behavior and response to medical and surgical treatment [2,3]. This narrative review of 52 research papers, involving up to 21 African nations and centered on the impact of COVID-19 on cancer care, has shown that the coronavirus pandemic has upset the delivery of cancer services in low- and middle-income nations, especially in Africa [4-7]. It has impacted cancer prevention, screening, diagnosis, management, palliative care, and patients' follow-up [8,9]. These disruptions especially during the peak of the pandemic are due to a number of factors. First, oncology healthcare workers were reassigned to the COVID-19 forefront and many times, without adequate personal protective equipment (PPE). Furthermore, the closure of country borders and movement restrictions posed logistic constraints resulting in the dearth of medications and clinical supplies requisite to cancer care delivery. Lastly, many oncological patients were prevented from visiting health facilities due to heightened fear of the COVID-19 virus [10,11] especially because they are at high risk for the acute form of the viral illness and fatal outcomes from reduced immunity caused by the malignancy itself, or consequent to radiotherapy, chemotherapy, [12] and surgeries [13] used in the management of persons with cancer. Telemedicine, small unmanned aerial systems (sUAS), and home therapy [14-16] are the most significant alternative care methods implemented across Africa during the peak of the pandemic to facilitate cancer services. This review is aimed at critically examining the impacts of the coronavirus pandemic on oncological services and highlighting the alternative cancer care delivery pathways implemented in Africa. Real-world data on these relatively novel cancer care strategies and their success rate in Africa so far is limited. As there is a need to determine which of these strategies can be tailored to the continued delivery of cancer services within complex healthcare systems, we intend that this paper gears further research to detail undocumented alternative cancer care delivery tactics and their successes, pan-Africa.

## Review

Study methodology in brief

For this narrative review, PubMed and Google Scholar were used to search for literature centred on cancer care in Africa since the first wave of the coronavirus pandemic till date; November 2019 to June 2023. Our search strategy involved; (Cancer OR Malignancy OR Tumour OR Oncology) AND (Covid 19 OR Coronavirus OR SARS COV2 Infection) AND (Management OR Treatment OR Care) AND (Africa OR “each of the 54 African countries”). Literature satisfying the search strategy was selected and exported into Rayyan.ai (a systematic review software) for duplicate removal and screening. The screening was in two phases. The first phase involved screening of titles and abstracts, making sure that selected manuscripts matched the inclusion criteria of being human studies, centred on cancer care in Africa, published since the first wave of the Covid-19 pandemic and written in the English language. Exclusion criteria include animal studies, meta-analysis, abstract-only papers, conference presentations, papers not written in the English language and literature not related to Africa. The second phase involved screening of cancer care types, the pandemic's impact on oncological care and strategies devised to mitigate the impact of the pandemic on the care of persons with cancer. From the fully screened papers, data gathered include the first author, title of the study, year of publication, country of origin, type of medical care provided, effect of the pandemic on cancer care and mitigating strategies. With only 52 papers fully satisfying the screening process just 21 African nations were found to have been mentioned in the papers. One other piece of literature that was included in this study was a paper written by Joung et al. and published in March 2022 so as to provide a developed country’s perspective from which the African community can learn [17]. 

Impact on the oncological workforce and physician-to-patient interaction

Oncological services require vast medical personnel as well as clinician-patient interaction in making diagnoses, administration of therapy and treatment response follow-up; however, the COVID-19 pandemic disrupted these significantly. The pandemic worsened cancer care staff shortages; oncologists in Africa are meagre in number which is why they have a higher workload when compared to the world’s average [18,19]. As a result of fear of coming down with the virus, particularly in settings where there was the inadequacy of PPE (as was the state in Ghana oncology centres as of May 2020) the situation worsened as many of these staff failed to show up at work [20]. The mental health of many frontline health workers was also affected and this necessitated the enactment of mental health teams as reported in Zambia as of May 2020 [5]. During the same time period in Zambia, staff were categorised into Schedule A, B and C such that Schedule A staff had to be physically present at work because they are essential staff, ‘B’ had to stay at home but report on a rotational basis and ‘C’ had to work from home [5]. This system may have in itself been advantageous in that truly, work efficiency can be attained whilst being physically absent from one's workplace. In a qualitative survey of March 2020 involving eight oncologists in Nigeria, it was mentioned interestingly that physical attendance to work had stopped in order to prevent cancer care staff from becoming carriers of the virus infecting other patients - thus being a way of protecting their patients according to the Hippocratic oath [11]. In Kenya, the pressure on oncological staff became enormous as clinic load increased from the pre-pandemic figure of 100 patients daily to over 250 patients per day as of March 2021 - a situation brought forth when the travel restrictions were lifted in an attempt to sort out backlog caused by the pandemic’s lockdown [8]. This situation has inevitably led to workforce burnout since the same time period in Ghana as well [8]. Moreover, the pandemic hampered physician-patient interaction. During the heat of the pandemic in Tunisia for instance, the number of consultations lessened from about 2,700 in May 2019 to a little over 900 in the same period in year 2020 as a result of movement restrictions as well as patients' anxiety and hesitancy in attending their clinic appointments [21]. A paper originating from the University of Ibadan, Nigeria, in July 2020 and the University of Cape Town, South Africa, in January 2021 highlighted that the fear of being infected with the Covid-19 virus due to hospital staff being potential carriers as the main cause of patients anxiety stalling clinic attendance [11,16]. Similarly, in a Rwandan oncology centre, there was a drop of over a quarter in the sum total of new outpatient cases during the peak of the pandemic in comparison to the pre-coronavirus era [22].

Impact on cancer screening and prevention

Many African nations do not have organized surveillance programs for early cancer detection. Only a few have screening programs for cervical cancer and, even in those nations, the surveillance rate is poor, particularly in Central Africa where just 6% of the target population is reached [23]. The dire state of cancer screening and prevention in Africa was further worsened by the COVID-19 pandemic. For instance, Zimbabwe’s countrywide coronavirus movement regulations and social distancing rules put forward to curb the spread of the virus from reporting its first case in March 2020 till the beginning of its third wave in June 2021 resulted in the cessation of surveillance services and awareness programs for cancer [24]. As of April 2021, Uganda, which had already recorded 337 Covid-19-related deaths and 40,000 Covid-19 cases, had in a bid to tackle the pandemic through lockdown and movement restrictions, also caused impedance to cancer outreach programmes and screening services [4]. Ghana, which was cited as having the second-best testing capacity of 13,364 tests per million to a population of 31.13 million, having recorded 215 Covid-19 related deaths and 2270 Covid-19 cases also had breast and cervical cancer screening as well as colonoscopic screening for colorectal cancers suspended during the peak of the pandemic in 2020 [8]. To date, there is very limited data suggesting a full return of these countries to their pre-pandemic cancer prevention states. The World Health Organization’s (WHO) statement published during the first wave of the pandemic in March 2020 recommending temporary stoppage of vaccination campaigns and immunization services in low-resource coronavirus hotspots [25] in the heat of the pandemic is likely to have worsened vaccination services against infections such as human papillomavirus (HPV) and hepatitis B virus; these viruses have been implicated in the pathogenesis of cervical and hepatocellular cancers respectively [26]. The endemicity of these cancers in African countries over the past two decades has remained unvarying [23] and the situation is more likely than not, to have been exacerbated by the coronavirus pandemic. With only 14 nations having initiated HPV vaccination across Africa [27], the pandemic may have quashed the intentions of other countries planning to come on board. Although the pandemic has been noxious to cancer prevention in Africa, it is very likely that recent advancements in technology may be the antidote. Borrowing a leaf from Zambia's response to the Covid-19 pandemic wherein clinical meetings involving patients and different strata of the medical care workforce have been taking on virtual platforms and telephonic means since May 2020 to date [5], cancer screening can as well be aided by these tools. This however would require patients to be more involved in their cancer screening processes. For instance, self-breast and testicular exams, self-diagnosis of skin lesions with the use of skin digital devices and self-test kits for cervical cancers. The results of these tests and management plans can then be discussed over virtual platforms with the index patient and multidisciplinary team as needed. Surely, there are risks associated with depending on patients' results from self-testing as these may be inaccurate. That is why Information, Education and Communication (IEC) materials should be provided through all forms of mass media in appropriate languages for the select population [24]. As it is likely that it may take a while before the African community incorporates technology fully into their health care systems, the WHO’s technical guidance on how to restore and maintain screening services as highlighted in a paper of the University of Zimbabwe in June 2021 must be adhered to [24].

Impact on cancer diagnoses

Cancer outcomes in the affected individual are hugely dependent on the stage at which it is detected. Delays in cancer diagnosis forestall treatment commencement, thus worsening patient outcomes. Cancer becomes difficult to cure once local tissue invasion and metastatic disease have occurred and overall, portends increased cancer-related morbidity and mortality [28]. The apprehension associated with the possibility of being infected with coronavirus in health facilities has negatively impacted early diagnostic programs globally [29]. This impact was remarkable during the first wave of the pandemic in low-resource settings particularly in Africa due to poor infectious disease control policies deterring cancer patients from visiting healthcare facilities for fear of developing the severe form of the COVID-19 disease [10,11]. Also, during the initial phase of the COVID-19 pandemic, many medical personnel were hesitant in assuming daily duty on account of the non-availability of PPE required as a safety measure against the infection [16]. The economic recession experienced in Africa during the peak of the pandemic posed financial constraints to the provision and delivery of oncological diagnostic services [16]. This situation was the case in certain Ghanaian health facilities where, by 12th April 2020 with 408 cases and eight deaths, routine cancer diagnostic procedures had become hugely delayed [6], as was the rescheduling of oncological imaging services used for cancer diagnosis in a number of Nigerian hospitals by July 2020 [30]. Although these are facility-level reports, it is logical to surmise that these interruptions occurred on a national scale and across many African countries and as such, worsened the existing state of poor cancer prognosis on the continent from delay in tests and investigations used in the diagnosis of cancer.

Impact on medical care

Whilst chemotherapy and radiotherapy play a critical role in cancer care, the coronavirus pandemic has had a marked effect on these modes of care for patients with cancer. By May 2020, Western and South African oncologists had halted chemotherapy and started adjuvant therapy for many patients [20]. During the same time frame in Sudan, cancer centres suspended non-urgent intravenous chemotherapy and follow-up appointments and delayed treatment for newly referred cases except in the instance of an emergency [20]. The situation was similar for radiation therapy as many patients were unable to access this form of cancer care for various reasons [20]. For example, it was reported that patients with cancer of the cervix in Zambia were unable to attend their radiotherapy appointments in the month of June 2020 [30] because the country experienced difficulties in importing radioactive medicines at the time it imposed movement regulations and international borders closure as measures against the COVID-19 pandemic [16]. In Sudan, although radiotherapy services were continued in oncology centres as of May 2020, patients domiciled in remote locations far from these facilities found it difficult to travel to receive treatment due to movement restrictions [20]. Ghanian reviews show that by January 2021, patients who ought to be administered chemo-radiation therapy received only radiotherapy whilst new referrals for this form of treatment were attended to depending on the effect of delays in treatment on prognosis [16]. Hypo-fractionated radiotherapy, which was not widely practised prior to the coronavirus pandemic, also gained quantifiable acceptance during the first wave of the pandemic in order to reduce the frequency of hospital admissions [16,32]. 

Repercussions are bound to happen when patients with cancer are denied access to medical care. The Ghanian populace, for example, has a sect that believes that consumption of large volumes of alcohol and strong faith could prevent the virus from infecting them [6]; meaning, they could ignore social distancing measures as well as personal hygiene without having to worry about being infected with the virus. Cancer patients having this mindset become a risk to themselves and their communities. First, they are likely to become infected with the virus. Second, the co-occurrence of cancer and Covid-19 in a single person increases the chance of mortality. Third, the consumption of huge volumes of alcohol in an individual with both cancer and Covid-19 causes a geometric rise in the chance of death and fourth, they are likely to inadvertently infect other people. Still, in Ghana, many people have strong beliefs that their Covid-19 test samples are being taken for ritual purposes and this made it difficult to curb the virus at its onset as of April 2020 [6]. In Nigeria, strong traditional belief systems do exist as well. This was seen about two decades ago during the global polio eradication programme wherein many citizens avoided being vaccinated because they thought it would render them infertile, infect them with HIV and would cause them to develop cancer. If this belief was upheld at that time, it is logical to assume that there may be some cancer patients in Nigeria and other parts of Africa who may not have been tested nor vaccinated against the Covid-19 virus and, this may lead to increased morbidity and mortality [6]. Although many papers since the first case of the pandemic have given alternatives to standard chemotherapy and radiotherapy regimens [5], it is difficult to state whether the regimens used by oncologists in Africa to care for cancer is right for the continent as Africa has always had to rely on regimen recommendations from the developed countries. It is therefore important to use this opportunity to carry out research on what regimen is best for Africa and concurrently, adapt the regimen alongside basic epidemiological measures leveraging technology as much as possible. 

Impact on surgical care

Many cancers if detected early are hugely amenable to surgical procedures. Delay in cancer surgeries has a detrimental effect on cure [33] as there is a 6-13% increase in death risk if an oncological operative procedure is delayed by four weeks [34]. The COVID-19 pandemic has caused marked delays in cancer surgeries [16,35]. During the one-hour-long expert panel discussion which was held by oncologists in Nigeria on 30th March 2020, it was reported in a few African countries including Nigeria, that patients in stable state who had been booked, hospitalized and awaiting elective surgeries were discharged without having their surgeries done [11,36]. According to a research paper involving Sudan, it was stated that during the onset of the pandemic, by April 2020, elective cancer surgeries had been cancelled for up to 14 days as the country was trying to understand and adapt strategies to combat the coronavirus pandemic [20]. In South Africa, it was reported that as of July 2020 surgical procedures for oncological cases had been cancelled or reduced in about 30% of health facilities surveyed [37] and it followed forecasting from a modelling study that had predicted that close to 13,000 surgical procedures were likely to be cancelled weekly across the country, including those for cancers [38]. Still, in July 2020, it was advised in the Good Clinical Practice document issued by the Lagos University Teaching Hospital, Nigeria that gynaecological cancer patients who had been infected by coronavirus and awaiting operative procedures should have their surgeries delayed by up to 14 days in order for the patents to recover from their viral illness so as to avert fatal outcomes associated with the co-existence of both pathologies in a single individual [30] perioperatively. Although there were significant suspensions in the number of surgeries performed for cancers across Africa, by January 2021 [36], it was reported that in select countries on the continent, breast cancer surgeries were mostly not postponed but, only a few patients deferred their surgery as a result of coronavirus related anxiety [39]. A number of factors have been highlighted as the reason for delays in cancer surgical procedures during the coronavirus pandemic in Africa. This includes the non-availability of operating theatres, a shortfall in the number of qualified medical and surgical personnel [40] and financial constraints experienced by cancer patients who required funds to cover the cost of surgery during the pandemic [10]. According to a paper published in March 2020, cancer patients have to pay out-of-pocket to access cancer care despite the fact that it is quite expensive [39]. It is therefore expected that there would be increased morbidity and mortality due to further financial crises caused by the loss of livelihood as a result of patients having to stay at home during the Covid-19 lockdown and movement restrictions. 

Impact on palliative care

End-of-life care is frequently required in patients with terminal cancer disease in order to improve symptoms and quality of life without necessarily making attempts at curing the affected individual. There were reports of the COVID-19 pandemic causing disruption to palliative care, particularly during the peak of the pandemic in May 2020. This was the situation in Morocco as there was a postponement of anticancer palliative therapy for oncological patients infected with coronavirus until they had made full recovery, and in Western Africa, secondary-level chemotherapy for palliative care was brought to a halt [20]. Very little data exist concerning the return to proper palliative care since the lockdown was lifted across the continent. 

Impact on cancer care in children

Whilst children have a low predisposition to coming down with the coronavirus disease and its complications, the public health regulations that were put in place against the pandemic have had an attendant effect on the diagnosis and treatment of cancer amongst the pediatric populace in many African countries [41,42]. These have been worsened by inadequate financial allocations to healthcare, paucity of appropriately trained medical personnel, non-availability of laboratories, delay in cancer surgeries, rescheduling of radiotherapy, non-standard alteration to chemotherapy regimens due to shortages in anticancer medications, blood products scarcity, difficulty accessing health care, inability to afford investigations and treatments and attend follow-up [10,16,43]. Almost 60% of pediatric oncologists involved in a transverse study carried out from 1st to 15th May 2020, involving 15 countries spanning the five regions of Africa indicate that the coronavirus pandemic has had a deleterious effect on the management of the top six childhood malignancies indexed in the World Health Organizations Global Initiative for Childhood Cancers [10]. Since the onset of the pandemic, children have continued to suffer from advanced local and metastatic disease and quite a number of them have not been offered effective treatment to date [43].

Impact on medical supplies and cancer research

Many healthcare facilities in Africa rely on the importation of medical supplies to cater for the medical needs of their patients; however, the COVID-19 pandemic has had an inimical effect on the availability of these consumables. As almost all the medications used in Ghana are imported, there was an acute depletion of medications stock because of importation restraint from border closures as of April 2020 [6]. A similar situation was reported in Zambia as many women missed multiple radiotherapy sessions in June 2020 as a result of international movement restrictions and consequently, difficulty in bringing across international borders, essential anti-cancer supplies [31]. As a result of shortages in COVID-19 test kits and PPE especially during the first, second and third waves of the pandemic, it became a norm for oncological facilities to decline and defer the treatment of patients with cancer [31,44]. As anticancer medications acute depletion has caused treatment delays and cancellations, the long-term implication of this situation remains unknown to date and needs to be monitored [45]; however, countries may continue to battle anticancer medications exhaustion due to multiple fold influx of cancer patients requiring anticancer therapy since the lift of lockdown as was the case in a Kenyan paper published in March 2021 [8]. It is important to mention that South Africa which is among the top seven developed countries in Africa struggled a lot with making available anticancer therapeutics by July 2021 as it hugely relies on pharmaceutical ingredient production in China and medicines production and export from India [8]. This therefore ought to serve as a pivot for the African community to delve into anticancer therapeutics production for self-reliance. Besides the impact on medical supplies, the pandemic also undermined cancer research activities, particularly at its onset [45]. In Morocco for example, whilst enlisting of new research patients was rescheduled, students in pursuit of a doctorate degree as well as administrative personnel were urged to not come to work by June 2020 [46]. In many other parts of Africa, there was a shutdown of research sites and laboratories, discontinuation of clinical research projects, prioritization of care of patients with coronavirus over research by physicians and the re-distribution of resources meant for routine studies on health to the development of COVID-19 vaccines and therapeutics [47,48] since the first reported case of Covid-19 up until now. Whilst these are acute impacts of the pandemic on cancer research, long-term complications have been reported by a few authors. The most important of them is a prolongation of drug development timelines resulting in overall increased cancer-related morbidity and mortality [47]. Research is the backbone of modern advancements in health. It is therefore important for African governments to increase funding and support to academic and research institutions [48] in order to return to their pre-pandemic state and become globally competitive.

Prospects

Africa can borrow a leaf from the return-to-screen (RTS) study published in March 2022, regarded as one of the largest efforts the United States of America has made to bridge the Covid-19 related screening gap [17]. The authors of the paper performed a national audit by creating a network of country-wide local audits and have found that there has been a gross fall in the national cancer screening rate when compared to the pre-pandemic era. They have suggested that patient reminders, provider assessments and reductions in structural barriers are required to return the country to the pre-pandemic screening rate within six months. This means that not just Africa was hit by the pandemic but, developed countries inclusive of the United States of America as well. The difference is that the developed countries are making giant strides in returning to their pre-pandemic states. If Africa were to bridge the screening gap by towing the RTS pathway, the first step will be for cancer facilities pan-Africa to form a network and use the RTS tool to define what screening was pre-pandemic and compare it to screening now. Then, the next step should involve setting a time target to return to the pre-pandemic screening rate through the mentioned evidence-based interventions. Next would be to set further medium and long-term goals to achieve up to 90% screening and vaccination rates within a specified period. Although prevention is the rate-limiting step if a continent free of cancer is to be achieved, the continent must advance in its cancer care delivery strategy by seizing this opportunity brought by the COVID-19 pandemic for technological advancement in healthcare. It is delightful to see that during the peak of the pandemic, telemedicine outpatient consultation replaced physical face-to-face interactions [6,49,50] and a number of patients already on follow-up as well as those only requiring prescription in Nigeria, Ghana and Morocco were digitally attended to [6,11]. Furthermore, a report from Rwanda published in March 2021 showed that drones were used to supply medicines to persons with cancer in order to prevent discontinuation of treatment [51]. Lastly, in Ghana, unmanned aerial vehicles used in the distribution of vaccines prior to the COVID-19 era were used to convey specimens of bodily fluids and tissue during the heat of the pandemic from local hospitals to major stations in order to centralize testing [52]. This leapfrogging depicts the potentiality of a pandemic in revolutionizing health care, pan-Africa. The successes of these relatively novel methods of cancer care delivery on the continent and their cost-efficiency is yet to be known; however, they are likely to reduce oncological morbidity and mortality and allow patients with cancer to continue therapy from home where possible [53]. 

## Conclusions

Beyond an iota of doubt cancer and Covid-19 have, to date, continued to remain topical issues of discussion around the world and particularly in Africa. At various peaks and troughs of the pandemic, many cancer care staff have experienced burns outs, mental health challenges and did stop presenting to work, especially at the onset of the pandemic. Cancer screening services, which were barely existing pre-pandemic, have suffered further declines. During the initial waves of the pandemic, cancer care facilities and hospitals shut down as a result of government regulations resulting in difficulty accessing cancer diagnostics and surgeries with patients consequently seeking alternative cures. Anticancer medicines became extremely scarce due to overreliance on importation. Palliative care, research activities and the WHO-indexed childhood malignancies as well, took a hit. Lessons to be learnt include that whilst essential cancer care staff need to be physically present to attend to patients, others can remain effective by leveraging virtual platforms. Furthermore, the RTS pathway needs to be adopted to bridge the Covid-19-induced cancer screening gap and develop strategies to improve cancer prevention pan-Africa. Funding is required from the government to make cancer care facilities, diagnostics and research facilities globally competitive. Academic institutes have to increase their capacity for training healthcare staff including doctors, nurses, paramedics and other allied health professionals. Paediatric oncologists need to figure out a way to return to proper management of the six childhood malignancies indexed in the WHO Global Initiative for Childhood Cancers. Although the pandemic has been injurious to cancer prevention, diagnosis, treatment and research, alternatives such as telemedicine, the use of drones and home therapy have to an extent, been effectuated to continue care of patients with cancer in Africa. Even with a number of countries adopting these alternatives to cancer care delivery, it appears that the coronavirus disease may have had a multiplier effect on cancer-related morbidity and deaths so far. The occurrence of the Covid-19 pandemic has shown that there is much room for improvement in the healthcare systems of Africa. There is also a need to study further and follow the relatively new alternatives adapted to cancer care delivery on the continent so as to determine and allow for their sustenance.

## References

[1] Ngwa W, Addai BW, Adewole I (2022). Cancer in sub-Saharan Africa: a Lancet Oncology Commission. Lancet Oncol.

[2] Zingue S, Atenguena EO, Zingue LL, Tueche AB, Njamen D, Nkoum AB, Ndom P (2021). Epidemiological and clinical profile, and survival of patients followed for breast cancer between 2010 and 2015 at the Yaounde General Hospital, Cameroon. Pan Afr Med J.

[3] Ezenkwa US, Imam MI, Yusuf MO, Giade AS, Imoudu IA, Katagum DA, Audu BM (2023). Cancer histotypes and trends in Azare, Northeast Nigeria: impact of diagnostic support disparity in data reporting. Ecancermedicalscience.

[4] Henke O (2021). Cancer care in East Africa amidst the Covid-19 pandemic. Int J Cancer.

[5] Lombe DC, Mwaba CK, Msadabwe SC (2020). Zambia's National Cancer Centre response to the COVID-19 pandemic—an opportunity for improved care. Ecancermedicalscience.

[6] Kugbey N, Ohene-Oti N, Vanderpuye V (2020). COVID-19 and its ramifications for cancer patients in low-resource settings: Ghana as a case study. Ecancermedicalscience.

[7] Umar S, Chybisov A, McComb K (2022). COVID-19 and access to cancer care in Kenya: patient perspective. Int J Cancer.

[8] Grossheim L, Ruff P, Ngoma T (2021). Cancer and COVID-19 experiences at African cancer centers: the silver lining. JCO Glob Oncol.

[9] Joseph A, Olatosi B, Haider MR (2022). Patient’s perspective on the impact of Covid-19 on cancer treatment in Nigeria. JCO Glob Oncol.

[10] Traoré F, Couitchere L, Michon J, Hessissen L (2021). Patient management in pediatric oncology during the COVID-19 pandemic: Report from francophone Africa. Pediatr Blood Cancer.

[11] Olabumuyi AA, Ali-Gombe M, Biyi-Olutunde OA (2020). Oncology practice in the COVID-19 pandemic: a report of a Nigerian expert panel discussion (oncology care in Nigeria during the COVID-19 pandemic). Pan Afr Med J.

[12] Zuze T, Ellis GK, Kasonkanji E (2020). Modified EPOCH for high-risk non-Hodgkin lymphoma in sub-Saharan Africa. Cancer Med.

[13] Mangouka GL, Iroungou BA, Moussavou-Boundzanga P, Nzenze JR (2022). Severe COVID-19 in 2 kidney transplant patients in Gabon. Am J Case Rep.

[14] Ismaili N, El Majjaoui S (2020). Management of breast cancer during COVID-19 pandemic in Morocco. Breast J.

[15] Oualla K, Nouiakh L, Acharfi N, Amaadour L, Benbrahim Z, Arifi S, Mellas N (2020). How is Morocco reacting to COVID-19 crisis in anticancer centers?. Cancer Control.

[16] Nnaji CA, Moodley J (2021). Impact of the COVID-19 pandemic on cancer diagnosis, treatment and research in African health systems: a review of current evidence and contextual perspectives. Ecancermedicalscience.

[17] Joung RH, Nelson H, Mullett TW (2022). A national quality improvement study identifying and addressing cancer screening deficits due to the COVID-19 pandemic. Cancer.

[18] Onyeodi IA, Fagbenro GT, Bashir MA, Awofeso OM, Joseph AO (2022). The path to becoming a clinical or radiation oncologist in Nigeria. Ecancermedicalscience.

[19] Diop M (2019). The state of medical oncology in Senegal. Ecancermedicalscience.

[20] Vanderpuye V, Elhassan MM, Simonds H (2020). Preparedness for COVID-19 in the oncology community in Africa. Lancet Oncol.

[21] Yahyaoui Y, Ghodhbani Z, Hamdi A (2020). Suggestion of Tunisia's medical oncologist in the management of breast cancer during COVID-19 pandemic. J Oncol Pharm Pract.

[22] Habinshuti P, Nshimyiryo A, Fejfar DL (2022). Impact of COVID-19 on access to cancer care in Rwanda: a retrospective time-series study using electronic medical records data. BMJ Open.

[23] Yang L, Boily MC, Rönn MM (2023). Regional and country-level trends in cervical cancer screening coverage in sub-Saharan Africa: A systematic analysis of population-based surveys (2000-2020). PLoS Med.

[24] Murewanhema G (2021). The COVID-19 pandemic and its implications for cervical cancer treatment and prevention in Zimbabwe: perspectives and recommendations. Pan Afr Med J.

[25] Abbas K, Procter SR, van Zandvoort K (2020). Routine childhood immunisation during the COVID-19 pandemic in Africa: a benefit-risk analysis of health benefits versus excess risk of SARS-CoV-2 infection. Lancet Glob Health.

[26] Amponsah-Dacosta E (2021). Hepatitis B virus infection and hepatocellular carcinoma in sub-Saharan Africa: Implications for elimination of viral hepatitis by 2030?. World J Gastroenterol.

[27] Kuehn B (2019). Summit leaders urge greater HPV vaccination in Africa. JAMA.

[28] Boyle P, Ngoma T, Sullivan R, Brawley O (2019). Cancer in Africa: the way forward. Ecancermedicalscience.

[29] Van Wyk AC, De Jager LJ, Razack R, Van Wyk SS, Kleinhans W, Simonds HM, Schubert PT (2021). The initial impact of the COVID-19 pandemic on the diagnosis of new cancers at a large pathology laboratory in the public health sector, Western Cape Province, South Africa. S Afr Med J.

[30] Okunade KS, Okunowo AA, Ohazurike EO (2020). Good clinical practice advice for the management of patients with gynaecological cancer during the COVID-19 pandemic in Nigeria and other resource-constrained countries. ecancermedicalscience.

[31] Lombe D, Phiri M, Msadabwe S (2020). Negative impact of the COVID-19 pandemic on the management of cervical cancer patients in Zambia. Ecancermedicalscience.

[32] Kochbati L, Vanderpuye V, Moujahed R, Rejeb MB, Naimi Z, Olasinde T (2020). Cancer care and COVID-19: tailoring recommendations for the African radiation oncology context. Ecancermedicalscience.

[33] Pumpalova YS, Ayeni OA, Chen WC (2022). The impact of breast cancer treatment delays on survival among South African women. Oncologist.

[34] Wassie L, Tsega S, Melaku M, Aemro A (2023). Delayed treatment initiation and its associated factors among cancer patients at Northwest Amhara referral hospital oncology units: A cross-sectional study. Int J Afr Nurs Sci.

[35] Addai BW, Ngwa W (2021). COVID-19 and cancer in Africa. Science.

[36] Lakhdar F, Benzagmout M (2020). Letter: Neurosurgery at war with the COVID-19 pandemic: patient's management from an African neurosurgical center. Acta Neurochir (Wien).

[37] Chu KM, Smith M, Steyn E, Goldberg P, Bougard H, Buccimazza I (2020). Changes in surgical practice in 85 South African hospitals during COVID-19 hard lockdown. S Afr Med J.

[38] COVIDSurg Collaborative (2020). Elective surgery cancellations due to the COVID-19 pandemic: global predictive modelling to inform surgical recovery plans. Br J Surg.

[39] Ayandipo O, Wone I, Kenu E (2020). Cancer ecosystem assessment in West Africa: health systems gaps to prevent and control cancers in three countries: Ghana, Nigeria and Senegal. Pan Afr Med J.

[40] Elkhouly EA, Salem RH, Haggag M (2020). Should cancer treatment be continued during the COVID-19 pandemic? A single Egyptian institution experience. Ecancermedicalscience.

[41] Marais BJ (2021). COVID-19 disease spectrum in children: first insights from Africa. Clin Infect Dis.

[42] Saab R, Obeid A, Gachi F (2020). Impact of the coronavirus disease 2019 (COVID-19) pandemic on pediatric oncology care in the Middle East, North Africa, and West Asia region: A report from the Pediatric Oncology East and Mediterranean (POEM) group. Cancer.

[43] Slone JS, Ozuah N, Wasswa P (2020). Caring for children with cancer in Africa during the COVID-19 crisis: implications and opportunities. Pediatr Hematol Oncol.

[44] Salako O, Okunade K, Allsop M (2020). Upheaval in cancer care during the COVID-19 outbreak. Ecancermedicalscience.

[45] Ezenwankwo EF, Nnaji CA, Moodley J (2022). Cancer service delivery and the impact of the COVID-19 pandemic in sub-Saharan Africa: a scoping review. Ecancermedicalscience.

[46] Mrabti H, Berrada N, Raiss G (2020). Cancer management challenge in a developing country in COVID-19 pandemic: reflection of a group of Moroccan oncologists. Future Oncol.

[47] Saini KS, de Las Heras B, de Castro J (2020). Effect of the COVID-19 pandemic on cancer treatment and research. Lancet Haematol.

[48] Kana MA, LaPorte R, Jaye A (2021). Africa's contribution to the science of the COVID-19/SARS-CoV-2 pandemic. BMJ Glob Health.

[49] Okeke M, Oderinde O, Liu L, Kabula D (2020). Oncology and COVID-19: Perspectives on cancer patients and oncologists in Africa. Ethics Med Public Health.

[50] Amaoui B, Lahlou L, Safini F, Semghouli S (2023). Teleconsultation use and satisfaction among cancerologists during the COVID-19 pandemic in Morocco. Pan Afr Med J.

[51] Martei YM, Rick TJ, Fadelu T (2021). Impact of COVID-19 on cancer care delivery in Africa: a cross-sectional survey of oncology providers in Africa. JCO Glob Oncol.

[52] Dalton M, Holzman E, Erwin E (2019). Patient navigation services for cancer care in low-and middle-income countries: A scoping review. PLoS One.

[53] Getachew E, Adebeta T, Muzazu SG (2023). Digital health in the era of COVID-19: Reshaping the next generation of healthcare. Front Public Health.

